# Assessing digital factors influencing AI-led asset information management in hospital facilities

**DOI:** 10.3389/fdgth.2026.1900969

**Published:** 2026-10-07

**Authors:** Motheo Tjebane, Thabo Khafiso

**Affiliations:** Department of Construction Management and Quantity Surveying, Faculty of Engineering and Built Environment, Durban University of Technology, Durban, KwaZulu-Natal, South Africa

**Keywords:** artificial intelligence (AI), asset information management, digital adoption factors, digital transformation, healthcare facilities

## Abstract

**Purpose:**

This study examines the digital factors influencing AI-led Asset Information Management (AIM) in hospital facilities. The study focuses on the extent to which digital factors related to data governance, operational efficiency, resource optimisation, and predictive capabilities are perceived to be present in healthcare facilities.

**Design/methodology/approach:**

A quantitative research approach was adopted using a structured questionnaire administered to relevant professionals involved in hospital asset management. Data were analysed using descriptive statistics in SPSS, including mean scores, standard deviation, skewness, and kurtosis to evaluate the distribution and significance of responses. Reliability testing was conducted, yielding a high Cronbach's Alpha value of 0.95, confirming strong internal consistency of the measurement instrument.

**Findings:**

A two-factor structure of digital factors was identified through an exploratory factor analysis. Although the statistical evidence points to a dominant one-factor structure, the two-factor solution was retained on theoretical grounds. Respondents rated foundational digital services, particularly access control and security protocols, mostly highly. One-way ANOVA comparing item-level mean scores across six groups of professionally experienced individuals showed no statistically significant differences. Suggesting that perceptions of these digital factors do not vary meaningfully by years of professional experience in this sample.

**Practical implications:**

The study highlights the need for healthcare institutions to invest in digital infrastructure, data integration, and capacity development to leverage AI capabilities fully. Emphasis should be placed on transitioning from basic automation toward predictive and intelligent asset management systems.

**Originality/value:**

This study provides empirical evidence on the layered adoption of AI in hospital asset management, demonstrating that current implementations are largely efficiency- and security-driven, while advanced predictive capabilities remain underdeveloped. It contributes to the growing body of knowledge by identifying a critical gap between AI potential and its practical realisation in healthcare infrastructure.

## 1 Introduction

Effective management of physical assets is a crucial component of infrastructure resilience, particularly in the healthcare sector. This is a sector where asset failure can directly impact patient safety and the quality of service delivery (Britton and Atkinson, 2017). Hospitals, characterised by complex building systems and life-critical medical technology, demand rigorous and proactive Asset Information Management (AIM) to ensure regulatory compliance, minimise costly downtime, and optimise maintenance expenditure (1). The shift from reactive breakdown maintenance to predictive maintenance has become a global operational imperative to sustain service continuity (2).

Despite this global shift, many public hospital systems in developing economies, such as South Africa, are constrained by systemic operational weaknesses (3). Current AIM practices are widely reported as fragmented, heavily reliant on error-prone manual processes, and plagued by severe data silos (4). This fragmented data ecosystem prevents a holistic view of asset health, resulting in ongoing maintenance backlogs, premature asset degradation, and significant long-term financial liabilities for the state (5). In essence, the existing infrastructure management paradigm is fundamentally incapable of meeting the demands of a modern, data-intensive hospital environment (6). Ajayi et al. (7) (2025) create an urgent need for models that can successfully integrate digital factors and artificial intelligence to drive predictive maintenance strategies.

While global scholarly discourse increasingly advocates deploying digital technologies, the comprehensive integration of AI within AIM often remains largely theoretical or focuses exclusively on contexts with abundant resources. Previous studies have examined the broader role of AI in optimising hospital operations and management. Scaife (8), systematically reviewed how AI and machine learning are being used to enhance predictive maintenance strategies. Click or tap here to enter text. Tjebane and Musonda (9) used a bibliometric and systematic review assessing the status and trends of AI applications in AIM within healthcare facilities. Even with these studies, there remains a critical gap in contextualising the barriers and enablers of AI-led AIM in developing economies such as South Africa. This creates a significant twofold gap between practical application and academic research.

In addition, a critical gap remains in contextualising the barriers and enablers of AI-led AIM within developing economies, such as South Africa (10). Patel and Ragolane (11) note that the discrepancy in question has two aspects, encompassing both practical implementation and academic investigation. Significantly, there is a lack in the contextual framework due to existing AI and AIM models typically being based on assumptions of abundant (12), high-quality (13), and integrated data streams (14), which contradict the actual conditions of the South African public hospital environment. Here, the AIM is constrained by resource limitations, infrastructural challenges such as aged infrastructure (15) and disparate legacy systems, as well as operational complexities. The current AIM is typically fragmented, heavily manual, and severely plagued by data silos (16).

A technological capacity gap exists, where traditional AIM systems lack the computational power required to process the large and diverse data streams essential for effective predictive maintenance (17). There is also a gap in the contextual framework, characterised by a limited empirical framework that specifically utilises AI techniques while being adapted to the resource limitations, infrastructural challenges, and operational complexities of South African hospitals. As a result of the knowledge gap, current interventions are insufficient to provide a practical, scalable roadmap for decision-makers seeking to shift from a reactive, manual management approach to a predictive, AI-driven system. In response to the identified empirical gap, this research aims to examine empirically the digital factors of AI-led AIM, which is explicitly designed to address the resource constraints and operational complexities characteristic of South African hospital facilities.

This study advances existing knowledge in multiple ways. It's unlike prior research on AI in health facilities, which was conducted in developed countries with abundant, integrated data infrastructure. This study also does not address AI adoption in hospital operations more broadly, but rather in asset information management, providing empirical survey-based evidence on digital factors. This study empirically distinguishes digital factors from advanced predictive and automation factors. Offering a more granular account of where AI-led AIM currently stands in this context than the largely conceptual or descriptive treatments found in South African studies (Nene and Hewitt, 2023). By testing whether perceptions of these digital factors differ by professional experience, the study offers an initial empirical basis, to be extended by future confirmatory and longitudinal research, for sequencing AI investment in public healthcare infrastructure.

## 2 Literature review

The successful integration of Artificial Intelligence (AI) into Asset Information Management (AIM) systems in hospital facilities is contingent upon the synergistic convergence of several digital factors (18). The core value proposition of AI in asset management lies in its capacity to transform heterogeneous operational data into actionable intelligence, thereby enabling the decisive transition from reactive to predictive maintenance paradigms (19). Hasan (20), notes of key requirement is that the system must consistently deliver accurate and timely data. This is to ensure that real-time, high-fidelity data throughput is essential for maintaining the integrity of the predictive models and ensuring that diagnostic outputs reflect the current operational status.

Crucially, contemporary maintenance data streams often comprise unstructured formats, including technician notes, audio logs, and thermal imagery. Therefore, effective AI must be able to effectively analyse unstructured data from maintenance informatics, converting qualitative information into quantitative inputs for risk modelling (21). By synthesising structured sensor data with unstructured inputs, AI can substantially enhance diagnostics of hospital facilities' assets, moving beyond simple fault detection to root-cause analysis and pattern recognition (22). This synthesis allows the system to accurately forecast potential issues before they escalate, facilitating the pre-emptive scheduling of interventions and minimising unplanned, catastrophic equipment downtime, which is particularly vital for life-critical assets in the hospital sector.

Automation is fundamental, as it shifts human focus from data entry and routine scheduling to complex problem-solving and strategic decision-making (23). One significant benefit (24) is the capacity to boost operational efficiency by automating repetitive tasks, such as work order generation, routine inspections, and low-level diagnostic checks. This automation concurrently streamlines workflow management, reducing manual intervention in standard processes —a mechanism critical in public hospital systems, which are often characterised by systemic administrative bottlenecks (3). AI-led automation plays a crucial role in ensuring the precise allocation of resources and personnel. By accurately forecasting asset failure and quantifying the required maintenance time, AI can optimise technician deployment and inventory staging, minimising wasted time and ensuring that skilled personnel are available when and where they are most needed (8). Among the most impactful specialised applications is the system's ability to manage the energy usage of the hospital facilities' assets (25). This AI-driven energy usage optimisation and management function is a high-value capability, as automatically adjusting operating parameters to reduce consumption without compromising asset performance or patient comfort contributes significantly to both sustainability and cost savings, representing a high-fidelity benchmark for advanced system maturity.

Volz et al. (2025) note that the integrity of the entire digital ecosystem is protected by data and security, with the constructs of data quality and security as non-negotiable prerequisites for success. For AI adoption in a risk-sensitive environment like healthcare, studies confirm that these foundational elements, particularly the enforcement of rigorous data security protocols and access controls, must be the priority. Robust data management underpins the accuracy of AI models, while stringent security protocols protect the highly sensitive nature of hospital operations data. Data integrity begins with ensuring that the system prioritises storing data relevant for future reporting and modelling. This involves not only collecting sensor readings but also tagging historical maintenance records and operational context, thereby forming a high-quality dataset necessary for training machine learning models (26). To combat the pervasive issue of data errors and fragmentation prevalent in developing economies, it is paramount that data validation and error correction procedures are used throughout all stages of data processing. This ensures the reliability of inputs and prevents the “garbage in, garbage out’ failure mode of AI systems (27). Serrano (2018) points out that modern AIM systems integrate physical infrastructure controls with digital information, security is of paramount importance. The system must use security protocols to safeguard sensitive information from unauthorised access and effectively manage access controls. It also needs to automatically adjust permissions based on needs, ensuring that only authorised personnel can interact with both the critical infrastructure controls and the underlying diagnostic data.

## 3 Methodology

### 3.1 Research design and approach

The research design employed a quantitative research approach, which was the most suitable methodology for achieving the research aim of empirically validating the digital driver (28). This approach involved collecting primary numerical data through a cross-sectional survey methodology. The quantitative paradigm was strategically selected because it facilitates the systematic testing of hypothesised cause-and-effect relationships between the latent digital factors (the independent variables) and operational effectiveness (the dependent variable) using inferential statistics (29, 30). Specifically, the design enables the generalisation of findings from the sampled population to the broader built-environment professional community within South African hospital facilities, thereby augmenting the framework's external validity. The cross-sectional design of the survey was chosen for its ability to efficiently collect data from a large, heterogeneous sample at a single point in time, which is adequate for mapping current adoption levels and testing the established conceptual relationships among the digital factor constructs. This design ensures the statistical inference required to move beyond descriptive analysis toward policy-relevant, evidence-based conclusions.

### 3.2 Target population and sampling technique

The Target Population for this study consisted of built environment professionals and facility managers who possess direct and active involvement in the management of hospital facilities, particularly those operating in digitalised environments within the South African context. Fahrenbach (2022) notes that this clear distinction was essential, as the concepts being examined require participants with extensive domain-specific knowledge and technical expertise to deliver dependable evaluations of present utilisation and potential viability. To ensure the participation of highly knowledgeable and domain-specific experts, who are necessary for assessing advanced technological constructs, a Judgmental (Purposive) Sampling technique was applied (31). This non-probability method was deemed essential given the specialised nature of the topic (Campbell et al., 2020), allowing the research to focus exclusively on individuals capable of providing the necessary depth of insight, thereby enhancing the validity and relevance of the empirical results (32). The distribution protocol systematically targeted professionals affiliated with recognised built environment professional bodies and relevant industry associations and networks to maximise the probability of obtaining responses from individuals with demonstrable expertise in both facility management and digital adoption. Following the rigorous distribution protocol, a total of 211 complete and usable responses were successfully procured. This sample size (*N* = 211) was confirmed to be statistically adequate for multivariate analysis techniques, such as Exploratory Factor Analysis and Analysis of Variance, satisfying the minimum requirements often cited in the literature for a high degree of statistical power (33, 34). Response completeness was rigorously verified through initial data screening procedures, including checks for missing values, thereby ensuring the high integrity of the final dataset and mitigating potential measurement error.

### 3.3 Data collection instrument and validation

The Data Collection Instrument was a structured, closed-ended questionnaire administered as an MS Forms survey to maximise reach and streamline data capture. The questionnaire was divided into sections, with Section A, Demographics, including professional experience, serving as the Independent Grouping Variable for the ANOVA. The instrument was structured into distinct sections corresponding to the digital factor indicators, later reduced through exploratory factor analysis into a two-factor structure. All items were measured using a 5-point Likert scale (1 = Strongly Disagree, 5 = Strongly Agree) to capture the degree of respondent agreement or importance regarding the operational indicators of the constructs. This specific scale was chosen to balance discriminative power with ease of administration, thereby reducing respondent fatigue and minimising central tendency bias (35, 36). The deployment via the MS Forms online platform enabled a broad geographic reach among South African professionals. It ensured efficient, automated data capture directly into a spreadsheet, which significantly reduced subsequent data-entry errors. Before the main data collection phase, the questionnaire underwent stringent validation to ensure methodological robustness. This included a comprehensive pilot study involving 30 domain experts to test the instrument's clarity, estimated completion time, and overall respondent comprehension. The pilot phase was immediately followed by an expert review process, during which a formal assessment was conducted to ensure content validity. This is to provide a comprehensive coverage of the research objectives and constructs. This confirms the unambiguous phrasing and logical flow. Minor refinements to the item wording and scale descriptors were implemented based on feedback from the validation phase to guarantee the final instrument's reliability and enhance data quality.

### 3.4 Data analysis technique

The gathered data were subsequently subjected to a stringent, two-stage statistical analysis protocol, adopted from Finch (2025) and Adebowale et al. (2025). The analysis was utilising the Statistical Package for the Social Sciences (SPSS). The first stage involved an Exploratory Factor Analysis (EFA) using Principal Component Analysis (PCA) with Varimax rotation. Before EFA, the reliability of the measurement scale was rigorously assessed using internal consistency metrics, specifically Cronbach's Alpha and Composite Reliability. This is to ensure that the construct indicators demonstrated acceptable levels of dependability (*α* > 0.7) for statistical inference (37, 38). This phase was critical for achieving data reduction and identifying the underlying, empirically derived dimensions or components (drivers) within the set of digital factor variables. The Kaiser-Meyer-Olkin (KMO) measure and Bartlett's test of sphericity are crucial statistical tests used to evaluate the suitability of the data for factor analysis (39). The KMO measure assesses the suitability of the data for factor analysis, with a value above 0.6 suggesting that the sample data is adequate (40). Bartlett's test of sphericity determines whether the correlation matrix is an identity matrix. A *p*-value below 0.05 indicates that the variables are sufficiently correlated to warrant meaningful factor analysis (41, 42). The tests verify that the dataset is suitable for extracting underlying factors, a requirement for the validity of factor analysis outcomes.

The process of component extraction based on eigenvalues greater than one and the suppression of factor loadings below 0.5 is a common practice in exploratory factor analysis (EFA) (43, 44). This approach helps in confirming the structural integrity and conceptual boundaries of the factors by ensuring that only significant components are retained and that the factors are clearly defined. Following the identification of the empirically defined driver components, an inferential statistical test, Analysis of Variance (ANOVA), was conducted. The purpose of this test was to determine if the mean scores of the two validated digital factors (Factor 1 and Factor 2) were statistically different across the six professional experience groups (0 years, 1–5 years, 6–10 years, 11–15 years, 16–20 years, >20 years). The analysis was performed by comparing the factor scores against the mean score of the Operational Effectiveness section, allowing the study to assess if differing levels of exposure and experience with AIM technology resulted in a statistically measurable difference in perceived operational outcomes (45).

### 3.5 Ethical considerations

The study strictly adhered to all ethical protocols mandated by the Institutional Research Ethics Committee (IR-EC) of the affiliated university. Formal ethical clearance and approval were secured before the commencement of any data collection activities. Participants were provided with comprehensive details regarding the study's objectives, the voluntary nature of their participation, and their unconditional right to withdraw at any stage without consequence. Informed consent was obtained electronically before granting access to the survey. Furthermore, all data collected was fully anonymised, with no personally identifiable information recorded, and data storage was governed by strict confidentiality protocols to ensure compliance with relevant national data protection legislation.

## 4 Findings

### 4.1 Demographics

This section outlines the demographic information of the respondents which covers the professional background, years of experience

#### 4.1.1 Profession

The study utilised a quantitative survey distributed to professionals in the built environment who are involved in hospital facility management, yielding a total of 211 responses. The occupational profile of the participants was heavily concentrated in core infrastructure and financial roles. The largest single group was Quantity Surveyors at 17.8%, followed closely by Construction Manager (15.2%), Electrical Engineer (14.7%), and Civil Engineer (12.6%), which together account for over 60% of the sample, indicating a strong foundation in the technical and commercial aspects of the built environment. Mid-tier representation included Construction Project Manager (9.9%) and Facilities Manager (8.9%), who are directly involved in asset oversight. Conversely, specialised roles such as Asset Manager (5.2%), IT and Network Specialist (5.2%), and Architecture (4.7%) were less represented, with the lowest percentages found in Operations Manager (3.1%) and Health, Safety, and Environmental Management Specialist (2.6%). This occupational mix demonstrates a sample drawn primarily from those professions responsible for the construction, engineering, and financial assessment of hospital assets, providing a relevant perspective for validating an AI-driven Asset Information Management (AIM) model.

#### 4.1.2 Years of experience

The professional experience profile of the sample indicates a substantial concentration of respondents in the early to mid-career stages. The majority of the 211 participants reported 15 years or less of experience, collectively comprising 85.2% of the total sample. Specifically, the largest single group had between 1 and 5 years of experience (37.4%), followed by those with 6 to 10 years (24.6%) and 11 to 15 years (18.5%), indicating that the findings are heavily weighted toward professionals currently engaged in or driving technological change. Only a small fraction of the sample consisted of highly experienced professionals with 16 to 20 years of service (13.3%) or more than 20 years (1.4%), while 4.7% reported no prior professional experience. This distribution is highly pertinent to the study's focus on developing an AI-led Asset Information Management model, as it captures the perspectives of the workforce most likely to implement and utilise such digital transformation initiatives

### 4.2 Descriptive statistics

#### 4.2.1 Factors influencing AI-led asset information management in hospital facilities

Table 1 provides a detailed statistical summary of the perceived presence of digital factors AI-led AIM indicators within South African hospital facilities, based on responses from *N* = 211 built environment professionals surveyed. The analysis employed a 5-point Likert scale, where 1 denoted Strongly Disagree and 5 denoted Strongly Agree. The overall descriptive results indicate a generally low-to-moderate level of digital factors. All measured indicators of the digital factors exhibited mean scores hovering slightly above or below the neutral point (3.0), indicating that no single AI-led AIM indicator currently achieves a high level of agreement or widespread institutionalisation. The internal consistency of the measurement scale was excellent, as indicated by the high Cronbach's Alpha = 0.95, which significantly exceeded the acceptable threshold of 0.7 for reliable statistical inference.

**Table 1:** Impacts of artificial intelligence on hospital asset management (source: authors).

Impacts of Artificial Intelligence on Hospital Asset Management	Mean	Std. Deviation	Skewness		Kurtosis	
			Statistic	Std. Error	Statistic	Std. Error
Manages access controls, automatically adjusting permissions based on needs	3.16	1.18	0.06	0.17	−0.87	0.33
Uses security protocols to safeguard sensitive information from unauthorised access.	3.16	1.15	−0.02	0.17	−0.63	0.33
Data validation and error correction procedures are employed throughout all stages of data processing.	3.13	1.10	0.11	0.17	−0.57	0.33
Manages the energy usage of the hospital facilities’ assets	3.13	1.23	0.04	0.17	−0.87	0.33
Streamlines workflow management, reducing manual intervention	3.12	1.18	0.11	0.17	−0.78	0.33
Ensures precise resource and personnel allocation	3.11	1.14	0.02	0.17	−0.73	0.33
Prioritises storing data relevant for future reporting	3.07	1.20	−0.04	0.17	−0.77	0.33
Effectively analyses unstructured data from maintenance informatics.	3.04	1.15	0.03	0.17	−0.68	0.33
Boosts operational efficiency by automating repetitive tasks	3.04	1.15	0.07	0.17	−0.62	0.33
Forecasts potential issues before they escalate	3.01	1.15	0.18	0.17	−0.62	0.33
Consistently delivers accurate and timely data.	2.99	1.10	0.05	0.17	−0.80	0.33
Enhances the diagnostics of hospital facilities’ assets	2.89	1.18	0.06	0.17	−0.69	0.33

Cronbach Alpha = 0.95.

The items with the highest mean score (3.16) were related to fundamental security and access control measures, specifically access controls and security protocols. This pattern suggests that the initial, moderate efforts towards digitisation in the facilities prioritise the non-negotiable prerequisites of protecting sensitive hospital data and controlling access. These are perceived as foundational and less complex capabilities compared to prognostic AI functions. Conversely, the lowest mean scores were observed for the core, high-value AI functionalities that are critical for achieving predictive asset management and enhanced decision-making. These include “Enhances diagnostics of hospital facilities’ assets” (Mean: 2.89) and “Consistently delivers accurate and timely data” (Mean: 2.99). This disparity highlights the Technological Capacity Gap: while the necessity for basic digital hygiene is moderately acknowledged, the implementation of advanced AI capabilities required for transforming data into actionable intelligence and enabling the shift from reactive to predictive maintenance remains the least realised aspect in the current hospital context.

The skewness and kurtosis values further support the interpretation of the moderate consensus. All skewness values fall within a narrow range (−0.04 to 0.18 with a standard error of 0.17), indicating a distribution that is close to symmetrical or only slightly skewed. The consistently negative kurtosis values (ranging from −0.57 to −0.87) suggest a platykurtic distribution. This implies that a substantial proportion of respondents clustered around the central, neutral point (3.0), reinforcing the interpretation that the digital factors have a low to moderate influence, with no strong institutional consensus yet reached on the widespread implementation of these digital factors.

#### 4.2.2 Factor analysis suitability

The appropriateness of the data for factor analysis was first assessed using the Kaiser-Meyer-Olkin (KMO) Measure of Sampling Adequacy and Bartlett's Test of Sphericity in Table 2. The KMO measure returned a value of 0.964, which is classified as excellent and significantly exceeds the recommended minimum threshold of 0.6 (Kaiser, 1974), confirming the high adequacy of the sample size and correlation matrix for factor analysis. Bartlett's Test of Sphericity was also highly statistically significant (chi2 = 2095.110, df = 66, Sig. = 0.000), rejecting the null hypothesis that the correlation matrix is an identity matrix and affirming that the variables are sufficiently correlated to warrant a meaningful EFA.

**Table 2:** Kaiser-Meyer-Olkin measure of sampling adequacy (source: authors).

Kaiser-Meyer-Olkin Measure of Sampling Adequacy.		0.964
Bartlett's Test of Sphericity	Approx. Chi-Square	2095.110
	df	66
	Sig.	0.000

#### 4.2.3 Total variance explained

The Initial Eigenvalues and Extracted Sums of Squared Loadings for the first two components are presented in the Table 3 below. The eigenvalue-greater-than-one criterion suggests a dominant single element (Factor 1) with an initial eigenvalue of 8.222, which accounts for 68.516% of the total variance. While the second component's eigenvalue (0.544) falls below the Kaiser criterion of 1.0, the retention of two factors aligns with the theoretical constructs established in the study's conceptual model. This indicates a strong unidimensional structure among the variables; however, the two-factor solution is maintained for theoretical consistency and the interpretation of the subdomains of digital factors.

**Table 3:** Eigenvalues and extracted sums of squared loadings (source: authors).

Component	Initial Eigenvalues			Extraction Sums of Squared Loadings		
	Total	% of Variance	Cumulative %	Total	% of Variance	Cumulative %
1	8.222	68.516	68.516	8.222	68.516	68.516
2	0.544	4.533	73.050	0.544	4.533	73.050

The Scree plot in Figure 1 consistently suggested the retention of a single dominant component. The Scree plot clearly displayed an “elbow” or sharp break after the first factor, with subsequent factors falling into the “scree” area, confirming that the vast majority of the variance is explained by the initial component (68.516%). Despite this statistical guidance toward a single-factor solution, the study adopted a two-factor solution based on the strong theoretical grounding of the model constructs, which distinguishes between foundational and advanced AI capabilities.

**Figure 1:**
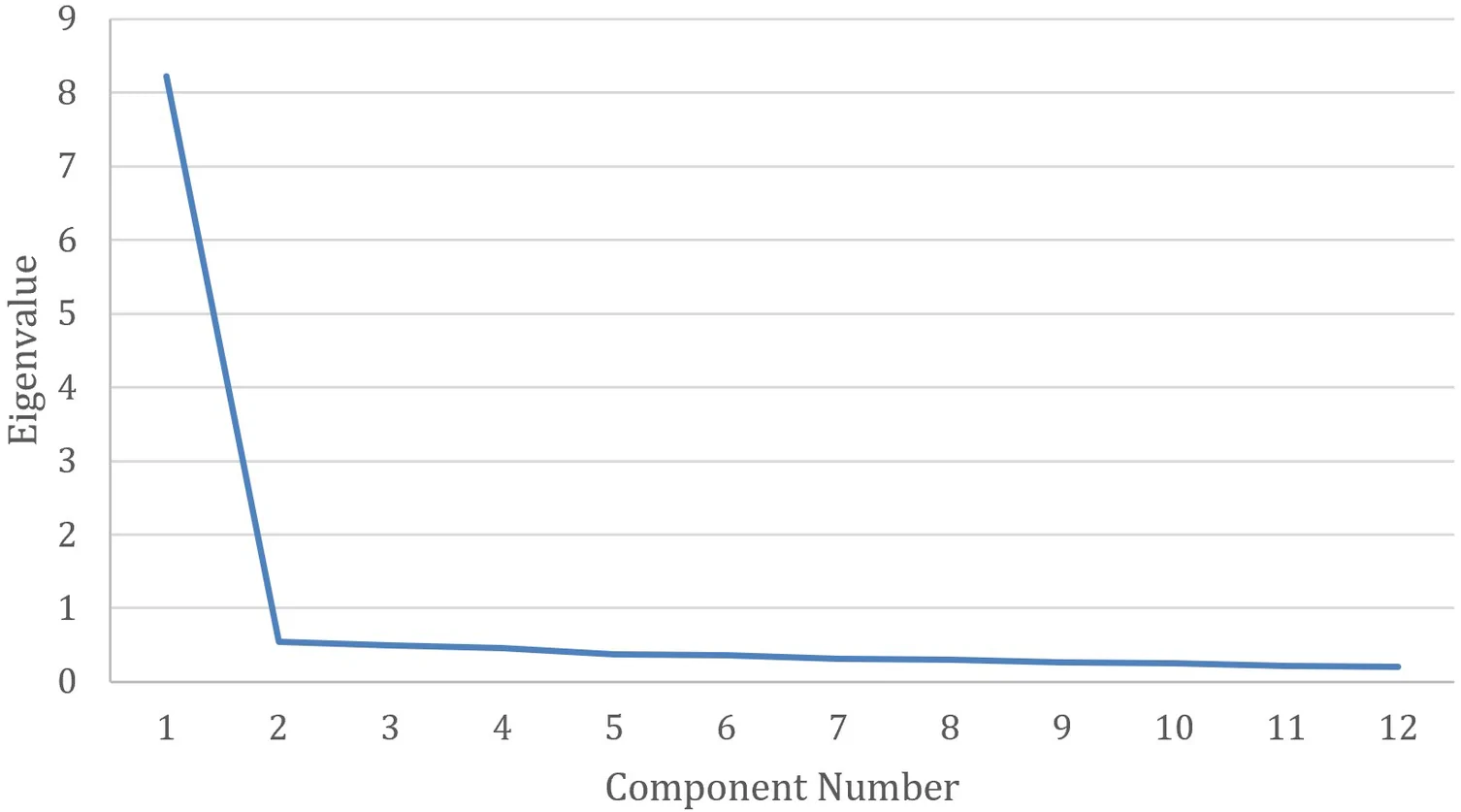
Scree plot (source authors).

#### 4.2.4 Component matrix and factor naming

The two-factor solution was retained on theoretical grounds rather than on the Kaiser eigenvalue-greater-than-one criterion, since Factor 2's eigenvalue (0.544) falls below this threshold and the scree plot supports a single dominant component. This is acknowledged as a methodological limitation of the present exploratory analysis (46). The retained second factor is best interpreted as a theoretically-informed subdomain within a predominantly unidimensional construct, rather than as a statistically independent factor, and this interpretation is offered with appropriate caution (47). Factor 1, “Integrated Data Management and Organisational Alignment,” accounted for a dominant 68.516% of the total variance explained by the model, indicating that the overall variability in respondents' perceptions of AI-led AIM digital factors is overwhelmingly concentrated in the foundational and prerequisite elements; this finding forms the basis for the subsequent discussion of the Technological Capacity Gap. The final rotated component matrix nonetheless showed a discernible distinction between two conceptual item clusters, with items loading strongly on one factor and near zero or low on the other. Items with loadings of 0.4 or higher were considered indicators of that factor and were retained on this threshold together with their conceptual relevance to the factor's theme. Given the below-threshold eigenvalue for Factor 2, this two-factor structure should be treated as provisional; confirmatory factor analysis and parallel analysis on an independent sample are recommended before the structure is used for scale development or theory testing as illustrated in Table 4.

**Table 4:** Component matrix and factor naming (source: authors).

Components	Factor 1	Factor 2
Uses security protocols to safeguard sensitive information from unauthorised access.	0.805	
Prioritises storing data relevant for future reporting	0.755	
Consistently delivers accurate and timely data.	0.732	
Ensures precise resource and personnel allocation	0.728	
Enhances the diagnostics of hospital facilities’ assets	0.704	
Boosts operational efficiency by automating repetitive tasks	0.666	
Manages access controls, automatically adjusting permissions based on needs	0.614	
Effectively analyses unstructured data from maintenance informatics.	0.428	
Data validation and error correction procedures are employed throughout all stages of data processing.	0.419	
Manages the energy usage of the hospital facilities’ assets		0.944
Forecasts potential issues before they escalate		0.542
Streamlines workflow management, reducing manual intervention		0.485

##### 4.2.4.1 Factor 1: integrated data management and organisational alignment

According to Table 5, this factor is defined by a strong clustering of items related to foundational data security, data quality, and resource governance. It represents the prerequisite infrastructure and management practices essential for any AI system to be reliable and trustworthy. The high loadings for security protocols (0.805) and data storage (0.755), alongside items related to resource allocation (0.728), confirm that this factor encompasses the basic operational and protective measures required for effective Asset Information Management (AIM). The lower-loading items concerning data validation (0.419) and unstructured data analysis (0.428) are retained here due to their direct conceptual link to data management quality.

**Table 5:** One-way ANOVA results for factor 1 items across respondent groups (source: authors).

Factor 1	Factors		Sum of Squares	df	Mean Square	F	Sig.
	Uses security protocols to safeguard sensitive information from unauthorised access.	Between Groups	5.65	5.00	1.13	0.85	0.52
		Within Groups	272.87	205.00	1.33		
		Total	278.52	210.00			
	Prioritises storing data relevant for future reporting	Between Groups	7.48	5.00	1.50	1.03	0.40
		Within Groups	296.45	205.00	1.45		
		Total	303.93	210.00			
	Consistently delivers accurate and timely data.	Between Groups	7.82	5.00	1.56	1.30	0.27
		Within Groups	247.13	205.00	1.21		
		Total	254.96	210.00			
	Ensures precise resource and personnel allocation	Between Groups	2.43	5.00	0.49	0.37	0.87
		Within Groups	272.07	205.00	1.33		
		Total	274.49	210.00			
	Enhances the diagnostics of hospital facilities’ assets	Between Groups	6.70	5.00	1.34	0.96	0.45
		Within Groups	287.79	205.00	1.40		
		Total	294.49	210.00			
	Boosts operational efficiency by automating repetitive tasks	Between Groups	8.15	5.00	1.63	1.23	0.29
		Within Groups	270.47	205.00	1.32		
		Total	278.62	210.00			
	Manages access controls, automatically adjusting permissions based on needs	Between Groups	4.33	5.00	0.87	0.62	0.68
		Within Groups	286.19	205.00	1.40		
		Total	290.52	210.00			
	Effectively analyses unstructured data from maintenance informatics.	Between Groups	11.65	5.00	2.33	1.79	0.12
		Within Groups	266.97	205.00	1.30		
		Total	278.62	210.00			
	Manages the energy usage of the hospital facilities’ assets	Between Groups	4.56	5.00	0.91	0.60	0.70
		Within Groups	310.99	205.00	1.52		
		Total	315.55	210.00			
Factor 2	Manages the energy usage of the hospital facilities’ assets	Between Groups	4.56	5.00	0.91	0.60	0.70
		Within Groups	310.99	205.00	1.52		
		Total	315.55	210.00			
	Forecasts potential issues before they escalate	Between Groups	3.11	5.00	0.62	0.47	0.80
		Within Groups	273.85	205.00	1.34		
		Total	276.96	210.00			
	Streamlines workflow management, reducing manual intervention	Between Groups	2.29	5.00	0.46	0.32	0.90
		Within Groups	290.51	205.00	1.42		
		Total	292.80	210.00			

##### 4.2.4.2 Factor 2: advanced predictive and process automation capabilities

This factor represents the higher-order, prognostic capabilities that distinguish a truly AI-led system. The item dominates the factor “*Manages the energy usage of the hospital facilities” assets”* (loading 0.944), which involves specialised, high-level automation. The inclusion of forecasted potential issues before they escalate (loading 0.542) confirms the factor's focus on predictive maintenance and future-oriented decision support. The item' Streamlines workflow management..' (loading 0.485) is conceptually retained, as it represents the core outcome of advanced process automation. This factor highlights the desired strategic shift from reactive to proactive asset management.

##### 4.2.4.3 ANOVA

The one-way ANOVA was conducted to test the null hypothesis that there is no significant difference in the mean scores of the individual digital factor items across the six professional experience groups. Specifically, the analysis aimed to determine whether greater experience with AIM (indicating higher exposure and potential adoption) was associated with a significantly higher mean score for perceived operational outcomes. The ANOVA results for all items are reported below. For a difference to be considered statistically significant, the Sig. Value must be less than 0.05. In this analysis, all Sig. Values are considerably higher, ranging from 0.12 to 0.90. This indicates no statistically significant differences in the perceived digital factor items by professional experience group for any item.

## 5 Discussion of findings

The findings in the study reveal various impacts of intelligence in the hospital management setting, which has illustrated a strong performance in the functionality of digital functionality, where hospital managers have improved access controls and automation based on the needs. Mehmood and Zafar (2026) and (48) assert that GenAI technologies introduce more benefits of asset management by enhancing automation, which also enhances the sustainability performance of the building, and offer data-driven decision-making to the asset managers. To further substantiate this finding, Pedral Sampaio et al. (2022) and (49) assert that through the Predictive nature of AI, the structural state of building services like sewer can be predicted around 66% to 75% and 71%, respectively, when compared with traditional models. Cheng et al. (2020) applied ANNs and SVM to predict the condition to maintain or repair mechanical, electrical, and plumbing (MEP) components and found around 96% and 97% of prediction accuracy. This shows that there is a huge impact that AI brings, especially in the structural performance perspective of the Hospitals, which is very crucial as they are mostly occupied most of the time.

A clear pattern that is emerging from the results is that AI has a big effect on data governance, cybersecurity, and access control systems. AI's ability to dynamically manage permissions and protect sensitive information shows how important it is for making sure that healthcare data is safe and compliant. This aligns with the literature, which emphasises that AI markedly improves data security, mitigates vulnerabilities, and fortifies governance frameworks in healthcare systems (Jiang et al., 2017; Reddy et al., 2020 and (50)). In hospitals, where asset data is often combined with patient information systems, these kinds of features are necessary to keep information private and operations running smoothly. The even distribution of responses also shows that institutions are aware of these benefits, which supports Topol's (2019) findings that the need to improve data security and trust often drives AI adoption in healthcare.

The results also show that AI plays a big role in data integrity and operational efficiency, especially when it comes to data validation, automating workflows, and making the most of resources. These features show that asset management is moving away from broken, manual methods and toward smart, integrated systems. The literature supports this change, saying that AI-driven automation lowers the risk of human error, speeds up processes, and helps hospital operations make better decisions (Davenport & Kalakota, 2019; Lee et al., 2021 and (51)). The inclusion of energy management as a key factor also shows how AI is becoming more common in sustainable healthcare infrastructure, where smart systems cut costs and use less energy (Zhang et al., 2022 and (52)). The differences in responses, on the other hand, suggest that the implementation wasn't consistent. This is in line with research showing that digital maturity varies widely among healthcare institutions, especially in developing countries (Marnewick & Marnewick, 2020).

Additionally, the findings underscore AI's role in facilitating data-driven decision-making and knowledge management, particularly through the storage of relevant data and the analysis of unstructured maintenance information. These capabilities are consistent with the literature on big data analytics in healthcare, which underscores AI's capacity to analyse extensive and intricate datasets to produce actionable insights (Wang et al., 2018). But the moderate placement of these factors shows that hospitals aren't making full use of these advanced analytical tools yet. Studies show that issues such as poor data quality, limited interoperability, and insufficient technical knowledge make it difficult for healthcare systems to use AI-driven analytics effectively (Krittanawong et al., 2021).

The results show that AI's ability to predict and diagnose problems is relatively weak. This includes predicting failures, ensuring real-time data accuracy, and improving asset diagnostics. These functions show how AI can transform operations by enabling predictive maintenance and proactive asset management. While the literature strongly supports AI's capability to deliver such outcomes (Baker et al., 2020; Esteva et al., 2019), it also highlights that these benefits are often constrained by inadequate infrastructure, insufficient data integration, and organisational resistance to change (Ranschaert et al., 2020). This elucidates the diminished emphasis noted in the findings, indicating that hospitals are still in the nascent phases of integrating advanced AI functionalities.

Two related questions warrant closer attention. Firstly, why are predictive functions comparatively lower-rated? Secondly, what does this mean specifically for the South African Health context? The lower means scores for diagnostic enhancement and real-time data accuracy are consistent with the technology capacity gap identified in this study. The healthcare-specific barriers documented in the South African context, such as funding constraints, aged buildings and disparate legacy systems, limit interoperability with new AI platforms and the shortage of digital skills among facilities staff (Nene and Hewitt, 2023; Chipps et al., 2025).

Beyond the technical barriers, organisational readiness, leadership commitment, change management capacity, and the financial investment required to move from pilot projects to institution-wide deployment are more likely to shape the extent to which foundational factors are more established than predictive ones (Bagherian and Sattari, 2022). Comparable patterns in which foundational digital hygiene precedes the adoption of advanced analytics have been reported in other developing-country healthcare and public-sector contexts (Bagherian and Sattari, 2022; Marnewick and Marnewick, 2020). Suggesting that the sequencing observed in this study may reflect a broader pattern of AI maturity in resource-constrained health systems rather than a South Africa-specific anomaly.

The statistical properties of the data bolster these interpretations. The near-zero skewness values indicate that the responses are evenly distributed, suggesting no significant bias. The negative kurtosis values show that the distribution is relatively flat, which means that respondents had different experiences. This pattern supports the idea that AI adoption is not uniform, since different institutions have different levels of digital maturity and ability to implement new technology. Similar findings have been documented in research investigating AI adoption in healthcare, emphasising variations in technological preparedness and resource accessibility among organisations (Nguyen et al., 2022).

## 6 Implications

The findings carry significant implications for theory and practice in resource-constrained environments.

### 6.1 Theoretical implications

The study presents an empirically derived, context-specific factor structure that extends existing AI adoption frameworks beyond developed-world contexts. By distinguishing between foundational (Factor 1) and advanced (Factor 2) digital factors, the research contributes a novel, granular understanding of the AI maturity curve in a public healthcare setting. The study also acknowledges that the two-factor structure requires confirmatory validation. The absence of statistically significant differences in perceived digital factor items across professional experience groups suggests that perceptions of AI-led Aim in this sample are broadly consistent. This is irrespective of experience level. This finding contributes to the theory by indicating that exposure alone may not be a strong driver of perceived maturity of digital factors in resource-constrained public healthcare settings. This proposition, however, was not directly tested and should be examined in future research.

### 6.2 Practical implications

The two-factor structure provides a preliminary, prioritised roadmap in Figure 2 for hospital administrators and government policy makers. Investment should be directed first toward consolidating integrated data management and organisational alignment. This includes mandatory protocols for data quality, digital security, and resource allocation planning. Without this foundation, the promise of advanced AI functionality is unlikely to be realised. Policymakers should prioritise the lower-scoring items identified in this study. Whether through targeted upskilling of maintenance staff and investment in data acquisition technologies. A phased implementation roadmap is proposed for AI-enabled predictive maintenance and energy management functions across facilities, moving fragmented pilots to institution-wide adoption as shown below.

**Figure 2:**
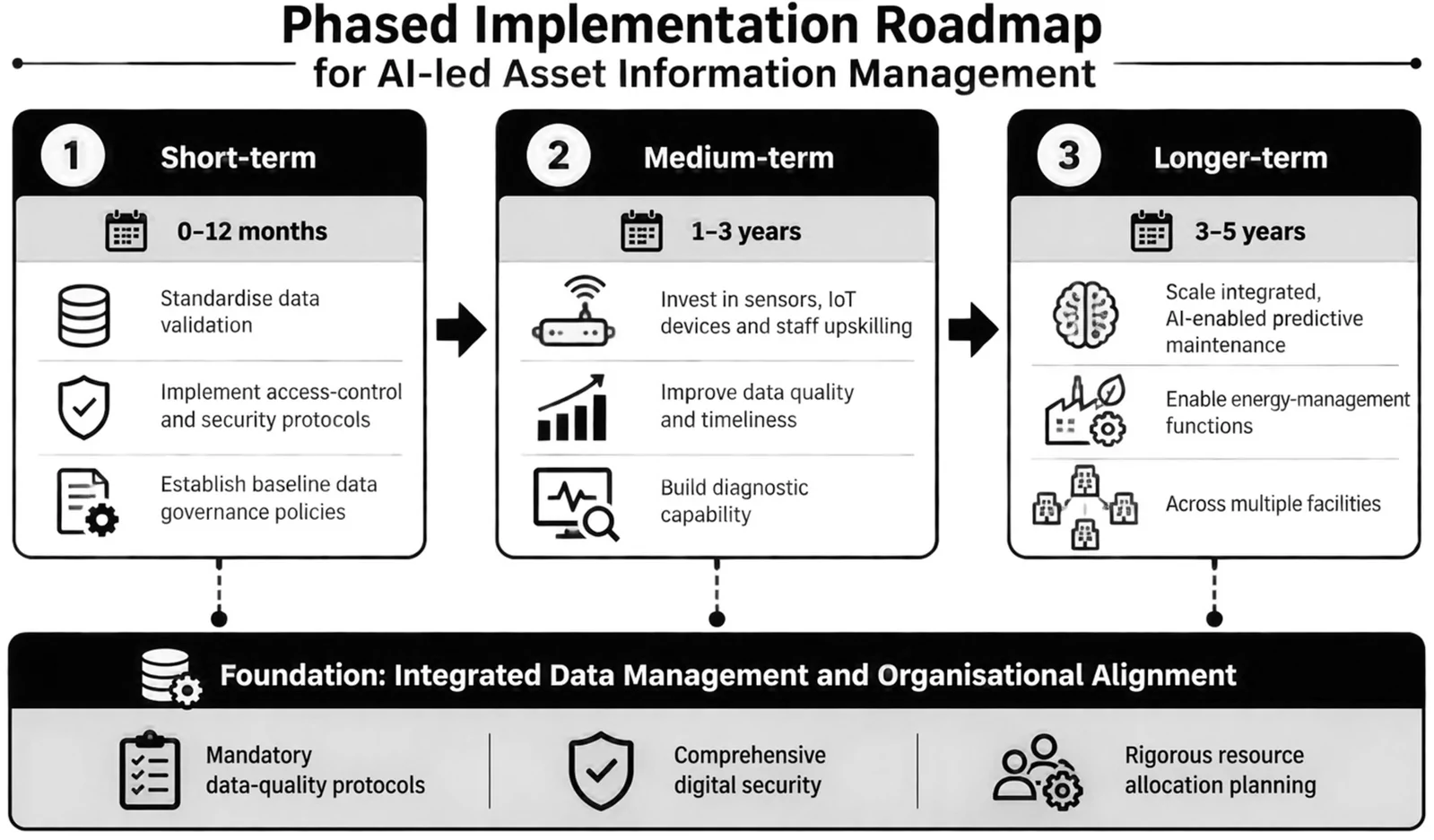
Phased roadmap (source authors).

### 6.3 AI governance and ethical considerations

The healthcare application context of this study makes AI governance an essential rather than a peripheral consideration. As hospital asset data is frequently interlinked with patient information systems, transparency, accountability, and cybersecurity of AI-enabled AIM platforms warrant explicit attention alongside their operational benefits. Responsible adoption requires clear governance structures for data privacy and access control, auditability of AI-driven maintenance recommendations and defined accountability for decisions made or informed by AI systems. Ethical oversight mechanisms, including institutional review of AI deployment and ongoing monitoring for unintended consequences, are recommended as hospitals progress from foundational to advanced ai led aim capabilities.

### 6.4 Interpreting the non-significant ANOVA results

The results from the One-Way Analysis of Variance (ANOVA), demonstrating that the mean scores of the two validated factors were not statistically significant across the six professional experience groups (*p*-value > 0.05 for all items), represent a key finding of this study. This non-significant result implies that, in their current state of adoption, the implementation of AI-led AIM factors has not yet yielded a statistically measurable positive effect on perceived Operational Effectiveness in the South African hospital context. This outcome is interpreted not as a failure of AI technology itself, but as empirical evidence that the adoption efforts are currently below a critical threshold of scale, maturity, and integration required to overcome the systemic operational variance prevalent in public facilities.

The descriptive results indicate that respondents are more aware of, or more confident in, foundational factors than in advanced predictive and automation capabilities. This pattern is consistent with the technological capacity gap identified in this study and suggests that, from the respondents' perspective. AI-led AIM in South African hospital facilities remains concentrated in foundational functions, with advanced predictive capabilities comparatively underdeveloped. Future research employing outcome measures and designs capable of testing causal or threshold relationships is needed to establish whether and how increased effort translates into measurable operational gains.

## 7 Limitations, recommendations for practice and future research

### 7.1 Limitations

This study has several limitations that should be considered when interpreting its findings. First, the cross-sectional design captures perceptions at a single point in time and does not permit causal inference; the reported associations, including the non-significant ANOVA results, describe perceptual patterns rather than causal or threshold effects. Second, the retained two-factor structure was justified primarily on theoretical grounds rather than being fully supported by the Kaiser criterion, and it has not yet been subjected to confirmatory factor analysis or replication on an independent sample. Third, the purposive sampling technique, while appropriate for accessing domain-specific expertise, limits the statistical generalisability of findings beyond the sampled population. Fourth, the findings are based on a single national context (South Africa); healthcare systems with different organisational structures, funding models, or levels of digital maturity may exhibit different patterns of AI-led AIM adoption, and the findings should not be assumed to be directly transferable to other contexts. Finally, self-reported perceptual data may be subject to social desirability or common-method bias, which future research employing objective performance indicators could help to address.

### 7.2 Recommendations for practice

Based on the empirical evidence, this study offers targeted recommendations for policymakers, hospital administrators, and built environment professionals. Strategic investment should focus first on strengthening Integrated Data Management and Organisational Alignment, including mandating robust data validation and error-correction procedures, establishing consistent data storage policies, and implementing comprehensive digital security frameworks, as prerequisites for the integrity of the input data that any predictive AI system would consume. Policy efforts should specifically target the lowest-scoring items related to data quality and diagnostics, through a concerted drive to invest in sensors, IoT devices, and focused staff training programmes to ensure that data is not only collected but is also accurate, timely, and suitable for advanced analysis. Policymakers should also consider moving from fragmented pilot projects toward more integrated, coordinated implementation across facilities, informed by the phased roadmap outlined in Section 6.2, while recognising that the operational benefits of such a shift remain to be empirically demonstrated.

### 7.3 Recommendations for future research

Several avenues for future research follow from this study's findings and limitations. Confirmatory factor analysis and parallel analysis on an independent sample are needed to test the robustness of the two-factor structure proposed here. Structural equation modelling or regression-based approaches, applied to a purpose-built operational effectiveness or performance outcome measure, would allow the causal relationships between digital factors and operational outcomes to be tested directly, which the present exploratory, cross-sectional design could not do. Longitudinal research is recommended to track how digital factor adoption and its perceived operational effects evolve over time. Comparative studies across different countries and healthcare systems would help establish the extent to which the findings generalise beyond the South African public hospital context. Case studies of hospitals that have successfully implemented AI-driven asset management, and mixed-methods research exploring organisational experiences during AI adoption, including resistance to change, digital skills gaps, and institutional culture, would complement the quantitative patterns identified here. Finally, future studies could examine the role of emerging technologies such as Digital Twins, the Internet of Things (IoT), Building Information Modelling (BIM), and Generative AI in hospital asset management.

## 8 Conclusion

This study empirically identified the digital factors influencing AI-led AIM in South African hospital facilities and described their current perceived state of adoption. The current level of AI-related digital factor adoption in South African hospital facilities is consistently low to moderate, with a clear pattern in which implementation is concentrated in non-negotiable prerequisites, such as security protocols and access controls, while core, value-adding AI functions critical for predictive maintenance, such as data quality and diagnostics enhancement, are the least realised. This suggests that the technologies necessary for advanced, strategic asset management are not yet fully mature in this context. The exploratory factor analysis identified a two-factor structure, comprising Factor 1: Integrated Data Management and Organisational Alignment and Factor 2: Advanced Predictive and Process Automation Capabilities. although the statistical dominance of Factor 1 and the below-threshold eigenvalue for Factor 2 mean this structure should be treated as provisional pending confirmatory validation. The one-way ANOVA found no statistically significant differences in perceived digital factor items across professional experience groups (*p* > 0.05 for all items), indicating that these perceptions are broadly consistent regardless of respondents' years of experience. Beyond its empirical findings, this study contributes a context-specific evidence base for a sector in which AI-led Asset Information Management is still emergent: by identifying where digital investment is concentrated and where critical gaps in predictive capability remain, it offers hospital administrators and policymakers a foundation for sequencing AI investment in ways that can strengthen operational resilience, support sustainable healthcare infrastructure, and, ultimately, safeguard the continuity and quality of patient care.

## Data Availability

The original contributions presented in the study are included in the article/Supplementary Material, further inquiries can be directed to the corresponding author.
